# Dazhu Hongjingtian Preparation as Adjuvant Therapy for Unstable Angina Pectoris: A Meta-Analysis of Randomized Controlled Trials

**DOI:** 10.3389/fphar.2020.00213

**Published:** 2020-03-10

**Authors:** Changfeng Man, Zhe Dai, Yu Fan

**Affiliations:** Institute of Molecular Biology and Translational Medicine, The Affiliated People's Hospital, Jiangsu University, Zhenjiang, China

**Keywords:** Dazhu hongjingtian, *Rhodiola wallichiana*, unstable angina pectoris, angina attacks, blood rheology, meta-analysis

## Abstract

**Objective:** Dazhu hongjingtian [DZHJT, *Rhodiola wallichiana* var. *cholaensis* (Praeger) S.H. Fu] preparation as an add-on therapy has been applied to the treatment of angina pectoris. We aimed to evaluate the efficacy and safety of DZHJT as adjuvant therapy for the treatment of unstable angina pectoris (UAP).

**Methods:** An extensive literature search was conducted on PubMed, Emase, Cochrane Library, Wanfang, CNKI, and VIP databases from inception to January 2019. Randomized controlled trials (RCTs) comparing DZHJT in combination with Western medicine with Western medicine alone were included. Two authors independently performed the literature search, data extraction and risk of bias assessment of included studies, and conducted the statistical analysis.

**Results:** A total of 18 RCTs involving 1,679 patients were included in the meta-analysis. Adjuvant treatment with DZHJT significantly decreased ≥80% reduction in the frequency of angina attacks [risk ratio (RR) 1.57; 95% CI 1.36–1.81], weekly frequency of angina attacks [mean difference (MD) −1.03 times; 95% confidence interval (CI) −1.51 to −0.55], marked improved abnormal electrocardiogram (RR 1.46; 95% CI 1.23–1.74). In addition, DZHJT significantly reduced the whole-blood viscosity (MD −0.70 mPa.s; 95% CI −0.84 to −0.55), plasma viscosity (MD −0.28 mPa.s; 95% CI −0.38 to −0.19), serum level of fibrinogen (MD −0.67 g/L; 95% CI −0.79 to −0.54), thromboxanes B2 (MD −14.01 ng/L; 95% CI −20.86 to −7.15), and C-reactive protein (MD −1.48 mg/L; 95% CI −2.72 to −0.25). No significant differences in headache/dizziness (RR 0.72; 95% CI 0.31–1.67) were observed between two groups.

**Conclusion:** Adjuvant treatment with DZHJT has an add-on effect in reducing angina pectoris attacks in patients with UAP. The beneficial effect may be correlated with regulating whole-blood viscosity, plasma viscosity, fibrinogen, thromboxanes B2, and CRP level. However, future well-designed prospective, randomized, double-blind placebo-controlled trials with large sample sizes are required to evaluate the evidence.

## Introduction

Angina pectoris is a symptomatic condition characterized by chest pain attacks. It is clinically classified into stable angina pectoris (SAP) and unstable angina pectoris (UAP). UAP is a type of acute coronary syndrome characterized by an attack at rest and severe, prolonged, and frequent or newly developed angina pectoris (Basra et al., 2016). The population weighted prevalence of UAP is 5.7% in men and 6.7% in women (Hemingway et al., 2008). UAP is associated with higher risk of acute myocardial infarction and sudden death. The current therapeutic strategy of angina pectoris mainly includes anti-ischemia, anti-thrombosis, and anti-platelet or revascularization procedures (Parikh and Kadowitz, 2014; Silva et al., 2015).

Dazhu hongjingtian (DZHJT)/*Rhodiola wallichiana* var. *cholaensis* [Praeger] S.H. Fu (R. wallichiana var.) has been frequently introduced to patients with angina pectoris in China (Fan et al., 2005). R. wallichiana var. is used for preparing DZHJT injection/capsule preparation, extracted from the root and rhizome. These preparations (detailed information of DZHJT is provided in Supplemental Text S1) have been approved by the Food and Drug Administration of China. Cardiovascular effects of DZHJT have been described in the dilation of cardiac vessels and reduction of myocardial oxygen consumption (Zhang et al., 2005). In addition, DZHJT also has anti-inflammatory activity (Choe et al., 2012), anti-diabetic effect (Gao et al., 2009), and sedative–hypnotic property (Li et al., 2007). Clinically, DZHJT is mainly used to treat angina pectoris (Jiang and Pan, 2012). A previous well-designed meta-analysis (Chu et al., 2014) has demonstrated the beneficial effects of DZHJT in SAP patients. Several clinical studies (Yu et al., 2011; Chen, 2013; Zhang, 2013; Cao et al., 2014; Jia and Wang, 2014; Li and Zhao, 2014; Shen et al., 2014) have investigated the add-on effects of the DZHJT in patients with UAP, but the findings were limited by small sample sizes and varying study quality. Therefore, we conducted this meta-analysis of randomized controlled trials (RCT) to assess the efficacy and safety of DZHJT as adjuvant therapy for patients with UAP.

## Materials and Methods

### Literature Search

We conducted this meta-analysis following the checklists of the Preferred Reporting Items for Systematic Reviews and Meta-Analyses Guidelines (Liberati et al., 2009). This meta-analysis was registered in the PROSPERO international database of prospectively registered systematic reviews (PROSPERO CRD42018111885). Two authors systematically searched PubMed, Embase, Cochrane Library, China Science and Technology Journal Database (VIP), China National Knowledge Infrastructure (CNKI), and Wanfang Database and from inception to January 2019. The searching items for English medical literature were “unstable angina pectoris” OR “angina” OR “acute coronary syndrome” AND “rhodiola” OR “hong jing tian” OR “hongjingtian” AND “randomized controlled trial” OR “randomized” OR “randomized.” Chinese searching terms included “bù wěn dìng xíng xin jiǎo tòng” OR “unstable angina pectoris” AND “hóng jing tiān” OR “rhodiola” AND “suí ji” AND “duìzhào.” A manual search was performed using the reference lists of relevant articles.

### Study Selection

Inclusion criteria were as follows: (1) study design was RCT; (2) patients diagnosed with UAP according to the guideline of the American College of Cardiology Foundation/American Heart Association (ACCF/AHA) (Braunwald et al., 2000), World Health Organization (Organization, 1979), European Society of Cardiology (ESC) (Fox et al., 2006) or Chinese Society of Cardiology (CSC) (Cardiology, 2000); (3) DZHJT in combination with conventional Western medicine vs. Western medicine alone; and (4) primary outcomes were ≥80% reduction in frequency of angina attacks weekly and marked improvement of abnormal electrocardiogram (restore normal or nearly normal defined by at least 0.05 mv restoration at ST segment). The secondary outcomes were the whole-blood viscosity, plasma viscosity, fibrinogen, thromboxanes B2, or C-reactive protein (CRP) and adverse events. Articles were excluded when: (1) diagnostic criteria for UAP were not specified; (2) patients have SAP; (3) combined application of DZHJT with other Chinese herbs as intervention.

### Data Extraction and Quality Assessment

For the included trials, two authors independently extracted the data and assessed the methodological quality. Any disagreements in this process were resolved by discussion. The extracted data included the last name of the first author, year of publication, sample size, patients' age, diagnostic criteria, interventions (dose of DZHJT and course of treatment), outcome measures, and methodological information. We evaluated the methodological quality of the included trials according to the Cochrane risk of bias tool, which included selection bias, performance bias, detection bias, attrition bias, reporting bias, and other sources of bias. Each trial was categorized by “high,” “unclear,” or “low” risk of bias.

### Statistical Analysis

The RevMan 5.2 software was used for the meta-analysis. We summarized as the risk ratio (RR) with 95% confidence intervals (CI) for dichotomous outcomes or mean difference (MD) with 95% CI for continuous outcomes. The Cochrane Q statistic and *I*^2^ index were applied to the analysis of heterogeneity across the studies. A random effect meta-analysis was conducted when the *p*-value of Cochrane Q statistic test is <0.10 and *I*^2^ >50%. Otherwise, we pooled the data by using a fixed-effect model. We used a funnel plot to examine the possible publication bias when the number of trials was sufficient. Leave-one-out sensitivity analysis was conducted to test the stability of the pooling results.

## Results

### Search Results and Study Characteristics

**Figure 1:**
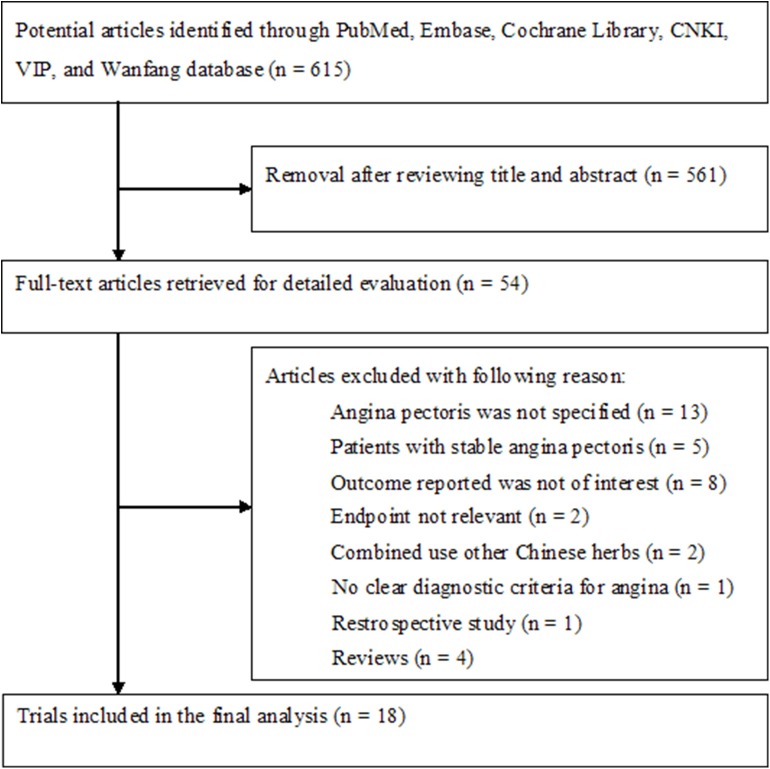
Flow chart of studies selection process.

In brief, our initial literature search yielded 615 potentially relevant articles. After screening the titles and abstracts, we retrieved 54 full-text articles for detailed evaluation. We further removed 36 articles on the basis of our predefined inclusion criteria. Thus, 18 articles (Yu et al., 2011; Chen, 2013; Zhang, 2013; Cao et al., 2014; Jia and Wang, 2014; Li and Zhao, 2014; Shen et al., 2014; Liu and Jiang, 2015; Wang et al., 2015; Weng et al., 2015; Zhai et al., 2015; Qin and Gao, 2016; Zhang and Lu, 2016; Du, 2017; Li, 2017; Li and Cheng, 2018; Wang and Yang, 2018; Zhang et al., 2018) were finally included in the meta-analysis (Figure 1).

The main characteristics of the included trials are summarized in Table 1. A total of 1,679 patients with UAP were identified with eligible trials. All of the selected trials were published in Chinese medical databases from 2011 to 2019. Two trials (Shen et al., 2014; Liu and Jiang, 2015) used DZHJT capsule as intervention, and the others used DZHJT injection. The duration of intervention ranged from 10 days to 8 weeks. Main conventional Western medicines referred to treatment included aspirin, nitrates, β-blockers, calcium channel blockers, angiotensin converting enzyme inhibitors, angiotensin receptor blockers, low molecular weight heparin, and lipid-lowering agents. All of the 18 trials indicated randomization, but only 4 trials (Chen, 2013; Liu and Jiang, 2015; Zhai et al., 2015; Zhang and Lu, 2016) described the detailed method of randomization. None of the trials reported the allocation concealment, dropout or withdrawal. Figure S1 shows the detailed methodological quality of the included trials.

**Table 1:** Baseline characteristics of the included trials.

**Study/year**	**No. patients DZHJT/Con**	**Age (years) DZHJT/Con**	**Diagnostic criteria**	**Main intervention**		**Treatment course**	**Outcome measures**
				**DZHJT group**	**Control group**		
Yu et al. (2011)	34/30	80–92	2000 CBCMA	DZHJT 10 ml/d, iv drop + control	Aspirin, trimetazidine, isosorbide dinitrate, and symptomatic treatment.	10 days	① + ⑧
Zhang (2013)	42/41	58.72 ± 12.86/60.72 ± 11.56	ESC	DZHJT 10 ml/d, iv drop + control	Aspirin, rosuvastatin, β-blockers, CCBs, and nitrates.	10 days	① + ③ + ④ + ⑧ + ⑧
Chen (2013)	30/30	61–84	2000 CBCMA	DZHJT 10 ml/d, iv drop + control	Aspirin, atorvastatin, β-blockers, nitrates, and ACEIs.	14 days	① + ② + ③ + ⑧
Li and Zhao (2014)	40/40	57.5 ± 5.6/58.1 ± 5.2	CBCMA	DZHJT 10 ml/d, iv drop + control	β-blockers, ACEIs/ARBs, nitrates, CCBs, and LMWH	15 days	③ + ④ + ⑤ + ⑥
Cao et al. (2014)	46/46	62–80	1979 WHO	DZHJT 20 ml/d, iv drop + control	ACEIs, β-blockers, antiplatelet, and lipid-lowering agents	14 days	①
Shen et al. (2014)	46/46	57.2 ± 8.1/58.2 ± 8.8	CBCMA	DZHJT capsule 5.56 g/d, po + control	Aspirin, metoprolol, enalapril, atorvastatin, and nitrates	8 weeks	② + ③ + ⑤ + ⑥ + ⑦
Jia and Wang (2014)	45/42	35–76	2000 CBCMA	DZHJT 10 ml/d, iv drop + control	Aspirin, statins, β-blockers, nitrates	10 days	① + ③
Liu and Jiang (2015)	40/40	56 ± 3/56 ± 4	CBCMA	DZHJT capsule 2.28 g/d, po + gf control	Aspirin, isosorbide dinitrate, and clopidogrel	8 weeks	① + ④ + ⑥
Weng et al. (2015)	61/62	66 ± 6/66 ± 8	2007 ACC/AHA	DZHJT 10 ml/d, iv drop + control	Aspirin, clopidogrel, nitrates, statins, and creatine phosphate sodium	10 days	② + ③ + ⑤ + ⑧
Zhai et al. (2015)	40/40	64.8 ± 2.3/60.2 ± 3.2	WHO	DZHJT 10 ml/d, iv drop + control	Aspirin, β-blockers, nitrates, statins, and creatine phosphate sodium	10 days	① + ② + ⑧
Wang et al. (2015)	40/40	39–75	CBCMA	DZHJT 10 ml/d, iv drop + control	Aspirin, atorvastatin, clopidogrel, metoprolol, isosorbide dinitrate, LMWH	14 days	② + ③
Zhang and Lu (2016)	27/27	60 ± 7/60 ± 8	2000 CBCMA	DZHJT 10 ml/d, iv drop + control	Aspirin, β-blockers, nitrates, statins, and clopidogrel	14 days	① + ② + ④ + ⑥
Qin and Gao (2016)	42/42	52–82	CBCMA	DZHJT 10 ml/d, iv drop + control	Aspirin, β-blockers, ACEIs/ARBs, nitrates, CCBs, and LMWH	10 days	⑧
Du (2017)	40/40	70.45 ± 9.83/71.02 ± 9.79	2000 CBCMA	DZHJT 10 ml/d, iv drop + control	Aspirin, trimetazidine, isosorbide dinitrate, and symptomatic treatment.	10 days	①
Li (2017)	39/39	57.75 ± 6.21/57.83 ± 6.07	CBCMA	DZHJT 10 ml/d, iv drop + control	Isosorbide dinitrate, statins, clopidogrel	14 days	① + ② + ③ + ⑦
Zhang et al. (2018)	63/63	60.3 ± 6.7	CBCMA	DZHJT 10 ml/d, iv drop + control	Anticoagulation, antiplatelet, antiischemia, salvianolate	14 days	④ + ⑥ + ⑧
Li and Cheng (2018)	38/38	58.21 ± 7.61/57.90 ± 7.04	CBCMA	DZHJT 10 ml/d, iv drop + control	β-blockers, antiplatelet, nitrates, CCBs	28 days	① + ⑦
Wang and Yang (2018)	130/130	49.3 ± 11.9/52.6 ± 10.3	CBCMA	DZHJT 10 ml/d, iv drop +control	Isosorbide dinitrate, statins, antiplatelet, and symptomatic treatment.	14 days	① + ③

DZHJT, Dazhu Hongjingtian; Con, control; ACEIs, angiotensin converting enzyme inhibitors; CCBs, calcium channel blockers; ARBs, angiotensin receptor blockers; CBCMA, Cardiovascular branch of Chinese Medical Association; ESC, European Society of Cardiology; ACC/AHA, American College of Cardiology Foundation/American Heart Association; ESC, European Society of Cardiology; LMWH, low molecular weight heparin.

① *≥80% reduction in frequency of angina attacks;* ② *weekly frequency of angina attacks;* ③ *marked improvement of abnormal electrocardiogram;* ④ *whole-blood viscosity;* ⑤ *plasma viscosity;* ⑥ *fibrinogen;* ⑦ *Thromboxanes B2;* ⑧ *adverse events*.

### Frequency of Angina Attacks

**Figure 2:**
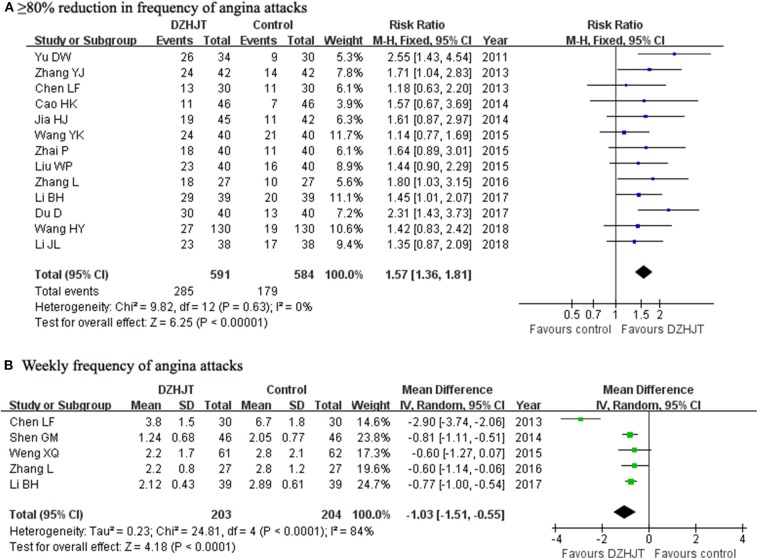
Forest plots showing comparison of ≥80% reduction in frequency of angina attacks **(A)** and weekly frequency of angina attacks **(B)** in patients with or without DZHJT treatment.

A total of 13 trials (Yu et al., 2011; Chen, 2013; Zhang, 2013; Cao et al., 2014; Jia and Wang, 2014; Liu and Jiang, 2015; Wang et al., 2015; Zhai et al., 2015; Zhang and Lu, 2016; Du, 2017; Li, 2017; Li and Cheng, 2018; Wang and Yang, 2018) selected ≥80% reduction in frequency of angina attacks as an outcome. As shown in Figure 2A, a fixed-effect model was applied because no heterogeneity was observed across trials (*I*^2^ = 0%, *p* = 0.63). Meta-analysis showed that adjuvant treatment with DZHJT significantly reduced the ≥80% reduction in frequency of angina attacks (RR 1.57; 95% CI 1.36–1.81). When we removed one trial (Yu et al., 2011) enrolling patients with age of more than 80 years, the pooled RR of ≥80% reduction in frequency of angina attacks was 1.52 (95% CI 1.31–1.76) in a fixed-effect model. Visual inspection of the funnel plot showed no evidence of publication bias (Figure S2). Five trials (Chen, 2013; Shen et al., 2014; Weng et al., 2015; Zhang and Lu, 2016; Li, 2017) reported the weekly frequency of angina attacks as an outcome measure. As shown in Figure 2B, a random effect model meta-analysis showed that adjuvant treatment with DZHJT was associated with a reduced weekly frequency of angina attacks [MD −1.03 times; 95% confidence interval (CI) −1.51 to −0.88; *I*^2^ = 84%, *p* < 0.001].

### Abnormal Electrocardiogram

**Figure 3:**
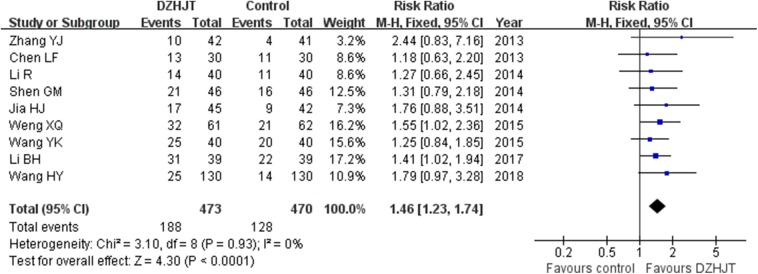
Forest plots showing comparison of marked improvement of abnormal electrocardiogram in patients with or without DZHJT treatment.

Nine trials (Chen, 2013; Zhang, 2013; Jia and Wang, 2014; Li and Zhao, 2014; Shen et al., 2014; Wang et al., 2015; Weng et al., 2015; Li, 2017; Wang and Yang, 2018) reported marked improvement of abnormal electrocardiogram as an outcome. As shown in Figure 3, a fixed-effect model meta-analysis indicated that adjuvant treatment with DZHJT was associated with marked improvement of abnormal electrocardiogram (RR 1.46; 95% CI 1.23–1.74; *I*^2^ = 0%, *p* = 0.93). No evidence of publication bias was observed based on the visual inspection of the funnel plot (Figure S3).

### Serum Fibrinogen, Whole-Blood Viscosity, and Plasma Viscosity

**Figure 4:**
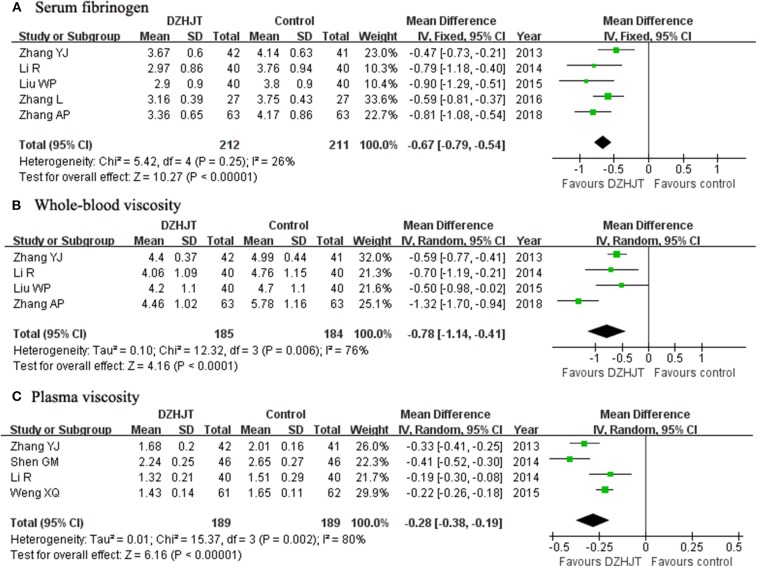
Forest plots showing comparison of the serum fibrinogen **(A)**, whole-blood viscosity **(B)**, and plasma viscosity **(C)** in patients with or without DZHJT treatment.

As shown in Figure 4A, a fixed-effect model meta-analysis of five trials (Zhang, 2013; Li and Zhao, 2014; Liu and Jiang, 2015; Zhang and Lu, 2016; Zhang et al., 2018) indicated that adjuvant treatment with DZHJT significantly reduced serum fibrinogen level (MD −0.67 g/L; 95% CI −0.79 to−0.54; *I*^2^ = 26%, *p* = 0.25). As shown in Figures 4B,C, a random effect model meta-analysis showed that whole-blood viscosity (MD −0.78 mPa.s; 95% CI −1.14 to −0.41; *I*^2^ = 76%, *p* = 0.006); four trials (Zhang, 2013; Li and Zhao, 2014; Liu and Jiang, 2015; Zhang et al., 2018) and plasma viscosity (MD −0.28 mPa.s; 95% CI −0.38 to −0.19; *I*^2^ = 80%, *p* = 0.002); four trials (Zhang, 2013; Li and Zhao, 2014; Shen et al., 2014; Weng et al., 2015) were significantly reduced in the DZHJT combined with Western medicine group.

### Serum Thromboxanes B2 and CRP Level

**Figure 5:**
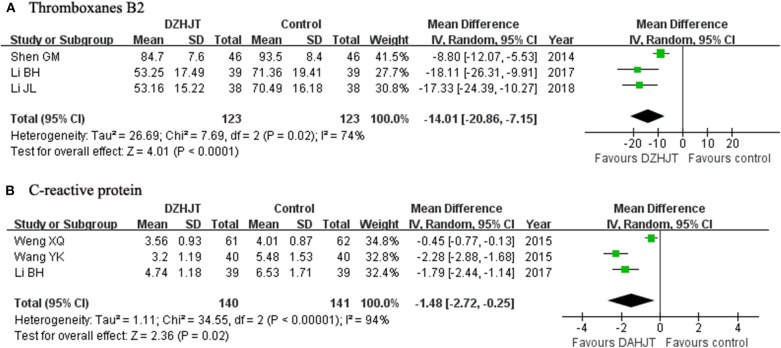
Forest plots showing comparison of the serum thromboxanes B2 **(A)** and C-reactive protein **(B)** level in patients with or without DZHJT treatment.

As shown in Figure 5A, a random effect model meta-analysis of three trials showed that DZHJT in combination with conventional Western medicine significantly decreased serum thromboxanes B2 level (MD −14.01 ng/L; 95% CI −20.86 to −7.15; *I*^2^ = 74%, *p* = 0.02); 3 trials (Shen et al., 2014; Li, 2017; Li and Cheng, 2018) compared with Western medicine alone. Moreover, Figure 5B shows that adjuvant treatment with DZHJT also significantly reduced serum CRP level (MD −1.48 mg/L; 95% CI −2.72 to −0.25; *I*^2^ = 94%, *p* < 0.001); three trials (Wang et al., 2015; Weng et al., 2015; Li, 2017) in a random effect model.

### Adverse Events

**Figure 6:**
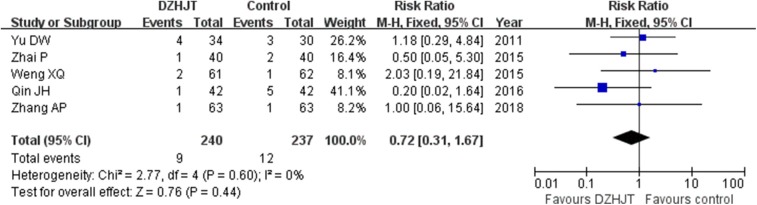
Forest plots showing comparison of incidence of headache/dizziness in patients with or without DZHJT treatment.

Five trials (Yu et al., 2011; Weng et al., 2015; Zhai et al., 2015; Qin and Gao, 2016; Zhang et al., 2018) described the adverse events. The common adverse events were headache and dizziness. No severe adverse events were reported. The incidences of headache and dizziness was 3.75 and 5.06%, respectively. As shown in Figure 6, no significant differences were found in headache and dizziness (RR 0.72; 95% CI 0.31–1.67; *I*^2^ = 0%, *p* = 0.60) between two groups. When we excluded one trial (Yu et al., 2011) enrolling patients with age of more than 80 years old, the pooled RR of headache and dizziness was 0.56 (95% CI 0.19–1.63) in a fixed-effect model.

## Discussion

The main findings of this meta-analysis suggested that adjuvant treatment with DZHJT significantly reduced the frequency of angina attacks and restored the abnormal electrocardiogram. Moreover, whole-blood viscosity, plasma viscosity, fibrinogen, thromboxanes B2, and CRP levels were significantly lower after DZHJT in combination with Western medicine treatment compared with conventional Western medicine alone.

Haemostatic parameters mainly include fibrinogen level, whole-blood viscosity, plasma viscosity, and hematocrit. These haemostatic parameters are elevated in patients with UAP (Neumann et al., 1991). Whole-blood viscosity represents the frictional resistance of blood flow on the intimal wall of blood vessels. Fibrinogen plays a major determinant in platelet aggregation and blood viscosity, whereas increased whole-blood viscosity may lead to high shear forces at the vascular endothelium, contributing to plaque instability (Cowan et al., 2012). Elevated haemorheological parameters correlate with the increased risk of cardiovascular events (Di Minno and Mancini, 1990; Lowe et al., 1997; Marton et al., 2003). DZHJT has the action of removing stasis and stopping bleeding. Therefore, it can reduce the high blood viscosity associated with blood stagnation. Our meta-analysis indicated that adjuvant treatment with DZHJT significantly decreased the whole-blood viscosity, plasma viscosity, fibrinogen, and thromboxanes B2 level. DZHJT significantly reduced serum CRP level. In summary, the beneficial effect of DZHJT in patients with UAP may correlate with the capability to normalize blood rheology and reduce the inflammatory reaction. However, whether DZHJT can decrease the development of coronary artery disease requires further investigation.

Most of the included trials did not select adverse events as outcome measures. None of the included trials reported severe adverse events. Headache and dizziness were the most frequently reported adverse events among these included trials. Headache may be more closely correlated with the use of nitrates (Thadani and Rodgers, 2006). Nevertheless, our pooled results revealed no significant differences in headache and dizziness between two groups. The possible adverse events associated with DZHJT use require further monitoring.

Several limitations in this meta-analysis must be noted. Firstly, the overall methodological quality of the included trials was suboptimal. All the included trials were generally of small sample size and none of the trials mentioned the sample size calculation, allocation concealment, and withdrawal/dropout or adopted the blinded, placebo controlled designs. Secondly, Traditional Chinese Medicine (TCM) is a holistic system of medicine. However, most of the included trials did not consider syndrome differentiation in patient selection. TCM syndrome differentiation must be incorporated into the diagnostic process and DZHJT is suitable for blood stagnation syndrome. Thirdly, generalizing the current findings to patients with SAP must be with caution. Finally, the included trials did not report the long-term follow-up results, and whether adjuvant treatment with DZHJT can reduce the risk of future cardiovascular events is unknown.

## Conclusions

This meta-analysis suggests that adjuvant treatment with DZHJT has an add-on effect in reducing the frequency of angina pectoris attacks among patients with UAP. The beneficial effect of DZHJT may be correlated with its function to regulate whole-blood viscosity, plasma viscosity, fibrinogen, thromboxanes B2 and CRP level. However, based on the existing evidence, no conclusion about the therapeutic benefits, limitations of use and potential risks can be drawn. Future well-designed prospective, randomized, double-blind placebo-controlled trials with large sample sizes are required to evaluate the evidence.

## Author Contributions

CM and ZD made the literature search, extracted data, evaluated the study quality, and performed the statistical analysis. CM drafted the manuscript. YF designed the study, interpreted the results, and revised the manuscript.

### Conflict of Interest

The authors declare that the research was conducted in the absence of any commercial or financial relationships that could be construed as a potential conflict of interest.

## Supplementary Material

Supplemental Text S1 The detailed information of DZHJT.

Figure S1 Risk of bias graph **(A)** and risk of bias summary **(B)**.

Figure S2 Funnel plots of trial reporting ≥80% reduction in frequency of angina attacks.

Figure S3 Funnel plots of trial reporting marked improvement of abnormal electrocardiogram.
