# Supplementary material for: Dazhu Hongjingtian Preparation as Adjuvant Therapy for Unstable Angina Pectoris: A Meta-Analysis of Randomized Controlled Trials
Source: Front Pharmacol. 2020 Mar 10;11:213. doi: 10.3389/fphar.2020.00213 (PMC7076193; doi:10.3389/fphar.2020.00213)
Supplement: Supplemental Text S1 — The detailed information of DZHJT. [file Data_Sheet_1.doc]

**Supplemental Text S1 The detailed information of DZHJT**

**1. Source**

DZHJT (*Rhodiola wallichiana*var. *cholaensis*[**Praeger**] **S.H. Fu**) belongs to the species from the family *Crassulaceae* in the genus *rhodiola*. About 90% of *Rhodiola* can be found in the northwest, southwest and northeast regions such as Tibet, Qinghai, Yunnan, Sichuan and other regions with high altitudes [1].

2. Main chemical **constitutes**

DZHJT contains gallic acid, 3,4‐dihydroxybenzoic acid, salidroside, p-coumaric acid-4-O-β-D-glucopyranoside, bergeninum, 4-hydroxybenzoic acid, 4-hydroxyphenylacetic acid, syringate, 6′′-O-galloylsalidroside, rhodiosin, rhodionin, kaempferol-7-O-α-L-rhamnoside, and many other compounds [1,2].

**3. Procedure**

Briefly, roots and rhizomes of Rhodiola sachalinensis were crushed into crude powder,and extracted under reflux with 70% ethanol. The combined extract was filtered, followed by ethanol reclamation through decompression, and concentrate to the ointments. The ointments were subjected to stationary and filtration for three times, then refrigerated with activated carbon. After removal of activated carbon, the supernatants were received the ultrafiltration, concentration, and freeze-drying.

**4. Quality control**

Salidroside is considered as one of the most effective components in DZHJT. Salidroside has been officially chosen as the quality control marker for DZHJT preparation, authorized by the China Drug and Food Administration [YBZ11852006]. For per milliliter DZHJT injection,this preparation at least contains salidroside 3.5mg and tyrosol 0.30mg. The qualitative analysis of the DZHJT was carried out by HPLC and LC-MS/MS(Chinese Pharmacopoeia 2015). As for DZHJT injection or capsule is the patented drug, quality control and chemical analysis of the material was not reported in the original trials.


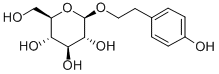

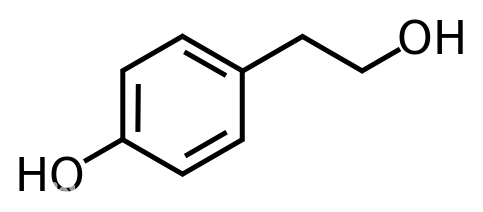


Salidroside molecular Formula: C_14_H_20_O_7_ Tyrosol molecular Formula: C_8_H_10_O_2_

**References**

[1] [Zhuang W](https://www.ncbi.nlm.nih.gov/pubmed/?term=Zhuang W%5BAuthor%5D&cauthor=true&cauthor_uid=30705774), [Yue L](https://www.ncbi.nlm.nih.gov/pubmed/?term=Yue L%5BAuthor%5D&cauthor=true&cauthor_uid=30705774), [Dang X](https://www.ncbi.nlm.nih.gov/pubmed/?term=Dang X%5BAuthor%5D&cauthor=true&cauthor_uid=30705774), [et](https://www.ncbi.nlm.nih.gov/pubmed/?term=Chen F%5BAuthor%5D&cauthor=true&cauthor_uid=30705774) al. Rosenroot (Rhodiola): Potential Applications in Aging-related Diseases. [Aging Dis.](https://www.ncbi.nlm.nih.gov/pubmed/?term=Rosenroot+(Rhodiola)%3A+Potential+Applications+in+Aging-related+Diseases) 2019;10:134-46.

[2] [Liu GD](https://www.ncbi.nlm.nih.gov/pubmed/?term=Liu GD%5BAuthor%5D&cauthor=true&cauthor_uid=27862112)2, [Zhao YW](https://www.ncbi.nlm.nih.gov/pubmed/?term=Zhao YW%5BAuthor%5D&cauthor=true&cauthor_uid=27862112), [Li YJ](https://www.ncbi.nlm.nih.gov/pubmed/?term=Li YJ%5BAuthor%5D&cauthor=true&cauthor_uid=27862112), et al. Qualitative and quantitative analysis of major constituents from Dazhu Hongjingtian capsule by UPLC/Q-TOF-MS/MS combined with UPLC/QQQ-MS/MS. [Biomed Chromatogr.](https://www.ncbi.nlm.nih.gov/pubmed/?term=Qualitative+and+quantitative+analysis+of+major+constituents+from+Dazhu+Hongjingtian+capsule+by+UPLC%2FQ‐TOF‐MS%2FMS+combined+with+UPLC%2FQQQ‐MS%2FMS) 2017;31(6). doi: 10.1002/bmc.3887.

[3] Wang XJ, Xie X,Luo X, et al. Chemical constituents from Rhodiola wallichiana var. cholaensis (I). Chinese Traditional and Herbal Drugs.2015;46:3471-4.
