# Supplementary figures and images for: Dazhu Hongjingtian Preparation as Adjuvant Therapy for Unstable Angina Pectoris: A Meta-Analysis of Randomized Controlled Trials
Source: Front Pharmacol. 2020 Mar 10;11:213. doi: 10.3389/fphar.2020.00213 (PMC7076193; doi:10.3389/fphar.2020.00213)

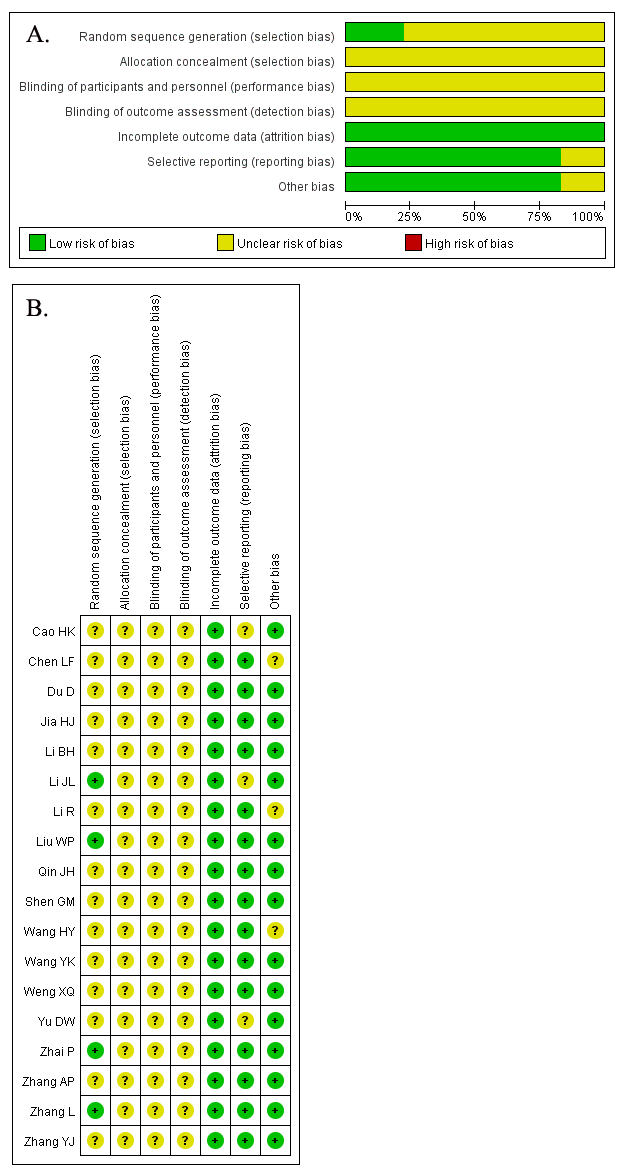

Supplement: Figure S1 — Risk of bias graph (A) and risk of bias summary (B). [file Image_1.TIF]

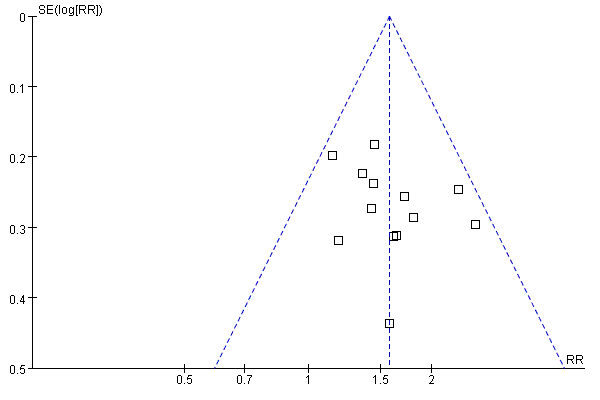

Supplement: Figure S2 — Funnel plots of trial reporting ≥80% reduction in frequency of angina attacks. [file Image_2.TIF]

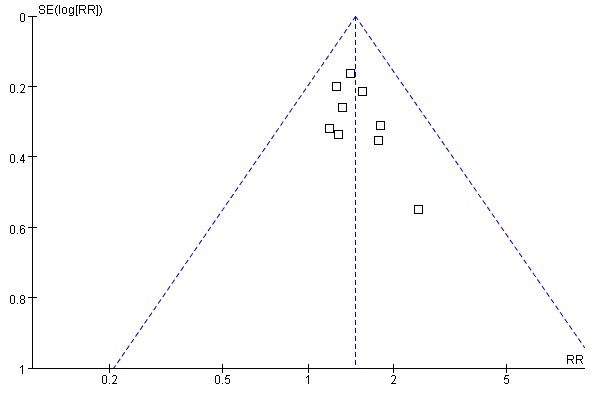

Supplement: Figure S3 — Funnel plots of trial reporting marked improvement of abnormal electrocardiogram. [file Image_3.TIF]
